# An Adaptive Virtual Impedance Method for Grid-Connected Current Quality Improvement of a Single-Phase Virtual Synchronous Generator under Distorted Grid Voltage

**DOI:** 10.3390/s23156857

**Published:** 2023-08-01

**Authors:** Caomao Zhong, Zhi Zhang, Anan Zhu, Benxin Liang

**Affiliations:** 1 Department of Electrical Engineering and Automation, Dongguan University of Technology, Dongguan 523808, China; 18162171778@163.com (C.Z.); 13712757251@163.com (A.Z.); 2 School of Automation, Guangdong University of Technology, Guangzhou 510006, China; lhj362358820@163.com

**Keywords:** VSG, weak grid, virtual impedance, harmonic suppression

## Abstract

The proportion of distributed generation systems in power grids is increasing, leading to the gradual emergence of weak grid characteristics. Moreover, using voltage-sourced grid-connected inverters can enhance the stability of a weak grid. However, due to the presence of background harmonics in weak grids, the grid voltage can cause significant distortions in the grid-connected current, which adversely affects the quality of the grid-connected current. This paper begins by briefly introducing the principle of the virtual synchronous generator (VSG). Then, the output current of the voltage source inverter is analyzed to elucidate the mechanism of harmonic current generation. Considering the distortion in the grid-connected current of the voltage source grid-connected inverter caused by background harmonics in the grid voltage, a harmonic current suppression strategy based on an adaptive virtual harmonic resistor is proposed. The proposed strategy employs a signal separation module based on multiple second-order generalized integrators connected through a cross-feedback network. This module effectively separates the fundamental and harmonic currents from the grid-connected current, extracts the amplitudes of the fundamental and harmonic currents through coordinate transformation, and adaptively adjusts the virtual harmonic resistance magnitude through the negative feedback control of the harmonic content (the ratio of the harmonic current amplitude to the fundamental current amplitude). These measures are used to enhance the quality of the grid-connected current. Additionally, the stability of the system is analyzed using the root locus of the open-loop transfer function. Finally, the effectiveness of the proposed method is validated through a combination of MATLAB/Simulink simulations and experimental results.

## 1. Introduction

In recent years, to address the global energy crisis, distributed power generation, represented by photovoltaic and wind power generation, has experienced rapid development [1]. Acting as an intermediary between distributed generation systems and power grids, grid-connected inverters play a pivotal role in converting the power generated by distributed sources into the AC form for transmission to the grids. It is essential for ensuring the safe and stable operation of power grids [2]. However, distributed generation systems based on inverter interfaces face challenges regarding participating in grid regulation due to their low rotational inertia and fast response [3]. Moreover, the integration of a significant number of connected distributed generation systems can adversely impact the stability of power systems [4]. Virtual synchronous generator (VSG) technology has attracted considerable attention from both domestic and international scholars [5,6]. This technology can be used to emulate the external characteristics of traditional generators, endowing grid-connected inverters with rotational inertia and damping characteristics similar to those of conventional generators. Due to the geographical dispersion of distributed generation systems, their connection to grids typically involves long-distance transmission lines. Consequently, grid impedance cannot be neglected, since it gradually manifests the characteristics of a weak grid [7,8]. In the presence of a weak grid, the point of common coupling (PCC) often experiences pronounced background harmonics [9]. These harmonics originate from the flow of nonlinear load currents through the grid impedance, resulting in substantial distortions in the grid-connected current of the grid-connected inverter [10]. This situation critically impacts the quality of grid-connected currents.

To ensure compliance with grid-connected standards, it is necessary to suppress the influence of background harmonics from grid voltages on grid-connected currents. In Reference [11], a multi-resonant compensator is added to the PR compensator. This enables the grid-connected current control loop to achieve high gain at the harmonic frequency, effectively suppressing harmonic currents. References [12,13] employ repetitive controllers as grid-connected current regulators to increase the gain at harmonic frequencies for achieving harmonic current suppression; however, designing regulator parameters is challenging. The use of a single repetitive controller negatively impacts system dynamic performance, prompting the combination of repetitive and PI control for improvement, as suggested in Reference [14]. In the suppression of harmonic currents, the grid voltage feedforward technique is widely employed and offers the effective suppression of background harmonics. The grid voltage proportional feedforward method proposed in Reference [15] demonstrates the superior suppression of low harmonics in grid-connected currents, although it proves less effective in suppressing higher harmonics. The grid voltage full feedforward control method, investigated in References [16,17], derives a comprehensive grid voltage feedforward function comprising proportional, primary differential, and secondary differential components. Compared to the grid voltage proportional feedforward approach, the grid voltage full feedforward method improves the suppression effect of the grid-connected inverter on higher harmonics. However, the differential component within the full feedforward function can amplify high-frequency signal interference. Voltage feedforward and full feedforward of grid voltage require the measurement of the voltage at the point of common coupling (PCC). However, in practical distributed generation systems, the lines are often long, making it difficult to measure the voltage at the PCC. One approach to address this issue is to measure it using additional communication lines, but this would increase the hardware cost. In Reference [18], a selective harmonic compensation scheme incorporating virtual impedance is employed for suppressing grid-connected harmonic currents. A thorough analysis of the Bode diagram and zero-pole diagram is conducted for three virtual impedances to ultimately select the virtual resistance as the optimal type of virtual impedance. Reference [19] proposes a Grid-side Current Harmonic Suppression (GCHS) method based on input voltage feedforward active damping. By extracting harmonic information and combining it with current setting and modulation voltage information, the suppression of current harmonics under different loads can be achieved. Reference [20] presents a novel full PCC voltage feedforward strategy for harmonic suppression in multi-parallel grid-tied inverters to reduce cost investments. The proposed strategy is based on adding virtual negative admittance to the target inverter to cancel the overall parallel admittance of the system. Reference [21] models the voltage deviation at the point of common coupling as an unmeasurable pseudo-disturbance and proposes a compensating disturbance observer to suppress harmonics based on this modeling. Table 1 shows a comparison of various control methods.

This paper presents a novel approach based on adaptive virtual harmonic impedance to address the existing issues in the aforementioned methods. It does not require measuring the voltage at the point of common coupling (PCC). Instead, it utilizes the algebraic virtual impedance method shown in Reference [22] to obtain the total voltage drop of the virtual impedance, which is then introduced into the dual-loop control. This method does not involve differential operations, thereby avoiding the problem of high-frequency signal amplification. In practical systems, low-order harmonics dominate the grid voltage, while high-order harmonics contribute only to a small extent. Therefore, the primary focus is on improving the grid current when there are variations in the content of low-order harmonics (third, fifth, and seventh harmonics) in the grid voltage. The adaptive virtual harmonic impedance introduces the harmonic content (expressed as the ratio of the harmonic amplitude to the fundamental frequency amplitude) into the double closed-loop control for negative feedback control of the harmonic content. By adaptively adjusting the magnitude of the virtual impedance based on the harmonic content in the grid-connected current, introducing the total voltage drop of the virtual impedance into the dual-loop control is equivalent to connecting a virtual harmonic resistor in series with the grid-connected system’s harmonic equivalent circuit. This reduces the harmonic current in the grid-connected current and thereby improves the quality of the grid-connected current.

## 2. Fundamental Principles of the VSG

**Figure 1:**
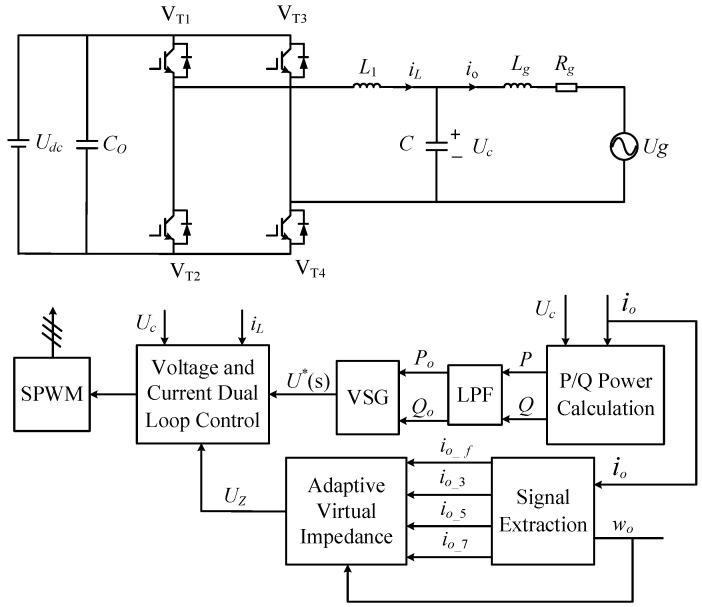
Main circuit topology and overall control block diagram of a single-phase VSG grid-connected inverter.

Figure 1 illustrates the main circuit topology and overall control block diagram of the single-phase VSG grid-connected inverter. The inverter-side inductor is denoted as *L*_1_, the filter capacitor as *C*, the grid-side inductor as *L*_g_, and the grid-side resistance as *R*_g_. The active power *P* and reactive power *Q* are calculated using instantaneous power theory [23]. However, due to the presence of secondary pulsations, a low-pass filter (LPF) is necessary to mitigate power oscillations and reduce disturbances.

The control algorithm for the VSG comprises active-frequency control and reactive-voltage control [24,25], and it can be expressed as follows:

(1) $$\frac{P_{m}-P_{e}}{w}=J\frac{dw}{\mathrm{dt}}+D(w-w_{ref})$$

(2) $$U_{o}=U_{ref}+k_{q}(Q_{ref}-Q_{o})$$

where *P_m_* and *P_e_* represent the mechanical and electromagnetic power of the VSG, respectively, while *w* and *w_ref_* denote the angular frequency of the VSG and the grid angular frequency, respectively. *J* and *D* represent the rotational inertia and damping coefficient of the VSG, respectively, and *Q_ref_* and *Q_o_* correspond to the reactive power reference and reactive power measured values, respectively. Similarly, *U_ref_* and *U_o_* represent the rated voltage amplitude and the actual output voltage amplitude, respectively. *k_q_* denotes the reactive power-voltage coefficient, where *P_e_* and *Q_o_* represent the instantaneous active and reactive powers obtained by the power calculation module. To more accurately simulate the characteristics of a synchronous generator, the primary frequency control is incorporated into the active power-frequency equation, as expressed in Equation (3). To enable the accurate tracking of the reactive power by the VSG inverter during grid connections, integral regulation is introduced to improve Equation (2), as shown in Equation (4).

(3) $$P_{m}=P_{ref}+\frac{w_{ref}-w}{k_{p}}$$

(4) $$U_{o}=U_{ref}+(k_{q}+\frac{k_{i}}{s})(Q_{ref}-Q_{o})$$

where *P_ref_* represents the active power reference value and *k_i_* is the integration coefficient.

**Figure 2:**
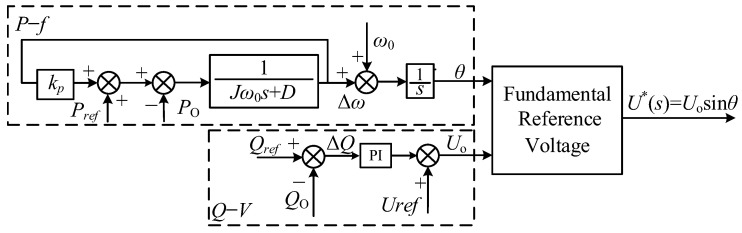
VSG control block diagram.

The control block diagram of the VSG is derived from Equations (1), (3) and (4), as shown in Figure 2. From Figure 2, it can be observed that the active power-frequency equation generates the reference frequency and phase, while the reactive power-voltage equation generates the reference voltage amplitude. The combination of these two equations forms the outer loop reference voltage for the voltage–current dual-loop control.

## 3. Analysis of the Harmonic Current Mechanism in Grid-Connected Voltage-Source Inverters under Non-Ideal Voltage Conditions

**Figure 3:**
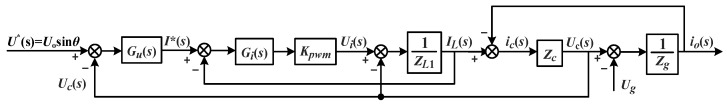
Equivalent model of voltage–current dual-loop control for VSGs.

The voltage–current dual-loop model of the VSG is illustrated in Figure 3. The outer loop, governed by the capacitor voltage, ensures voltage stability, while the inner loop, controlled by the inverter-side inductor current, achieves fast current tracking and enhances the system dynamic response speed. In this paper, the SPWM modulation strategy is adopted, with *K_pwm_* representing the equivalent gain of the inverter, set to a value of 1. Control is implemented on the *αβ* axis, where the inner loop current controller *G_i_*(s) employs proportional (*P*) control to track the current reference. Meanwhile, the voltage outer loop controller *G_u_*(*s*) utilizes proportional-resonant (*PR*) control. The transfer function of the PR controller is as follows:

(5) $$G_{u}(s)=k_{p}+\frac{2k_{f}w_{c}s}{s^{2}+2w_{c}s+w_{o}^{2}}+\sum_{h=3,5,7}\frac{2k_{h}w_{c}s}{s^{2}+2w_{c}s+{\left(hw_{o}\right)}^{2}}$$

where *k_p_* is the proportional gain, *k_f_* is the fundamental frequency gain, *k_h_* is the harmonic frequency gain, *w_c_* is the bandwidth of the controller, *w_o_* is the voltage fundamental corner frequency, and *h* represents the signal harmonic order.

**Figure 4:**
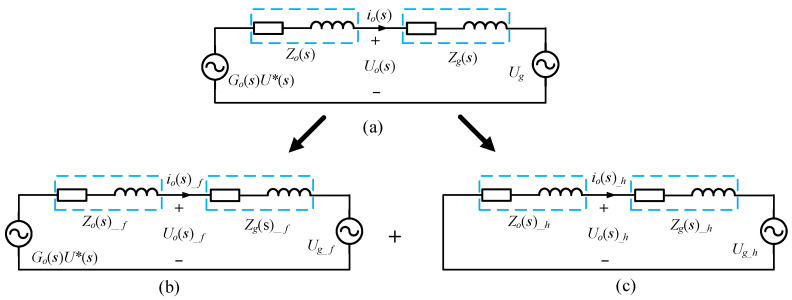
Equivalent circuit diagram of the grid-connected system. (**a**) Overall equivalent circuit for grid-connected systems. (**b**) Fundamental equivalent circuits for grid-connected systems. (**c**) Harmonic equivalent circuits for grid-connected systems.

According to Figure 3, it can be observed that the relationship between the reference voltage U*(*s*) of the outer loop and the inverter output voltage *U_c_*(*s*) and grid current *I_o_*(*s*) is given by Equation (6)

(6) $$U_{c}(s)=G_{o}(s)U^{*}(S)-Z_{o}(s)i_{o}(s)$$

where *G_o_*(*s*) and *Z_o_*(*s*) are, respectively,

(7) $$G_{o}(s)=\frac{G_{u}(s)G_{i}(s)K_{pwm}Z_{c}}{Z_{L1}+Z_{c}+G_{i}(s)K_{pwm}+G_{u}(s)G_{i}(s)K_{pwm}Z_{c}}$$

(8) $$Z_{o}(s)=\frac{Z_{L1}Z_{c}+G_{i}(s)K_{pwm}Z_{c}}{Z_{L1}+Z_{c}+G_{i}(s)K_{pwm}+G_{u}(s)G_{i}(s)K_{pwm}Z_{c}}$$

where *Z_L_*_1_ = *sL*_1_ and *Z_c_* = 1/*sc*. According to Equation (6), the grid-connected inverter can be equivalently represented as a voltage source *G_o_*(*s*)U*(*s*) in parallel with an equivalent output impedance *Z_o_*(*s*), while the weak grid can be equivalently represented as a series connection of the grid voltage *U_g_* and grid-side impedance *Z*_g_(*s*), where *Z*_g_(*s*) = *sL*_g_ + *R*_g_. Hence, the equivalent circuit of the grid-connected system can be obtained as shown in Figure 4.

The expression of grid-connected current *i_o_*(*s*) can be obtained from Figure 4 as follows

(9) $$i_{o}(s)=\frac{G_{o}(s)U^{*}(s)}{Z_{o}(s)+Z_{g}(s)}-\frac{U_{g}}{Z_{o}(s)+Z_{g}(s)}$$

According to Equation (9), the grid-connected current *I*_o_(*s*) is composed of two parts: the effect component from the outer loop reference voltage U*(*s*) and the disturbance component generated by the influence of the grid voltage U_g_. When the grid voltage *U_g_* contains low-order harmonics, the grid-connected current *I_o_*(*s*) will generate harmonics of the same order.

## 4. Adaptive Virtual Harmonic Resistance and Fundamental Reactance Algorithm

According to Equation (9), increasing the magnitude of *Z_o_*(*s*) + *Z*_g_(*s*) can suppress the influence of the grid voltage on the grid-connected current. However, *Z_g_*(*s*) represents the grid impedance, which is difficult to alter. Therefore, the improvement of the grid-connected current can only be achieved by changing the inverter output impedance *Z_o_*(*s*). By employing the method of virtual impedance, the magnitude of *Z_o_*(*s*) can be increased to reshape the inverter output impedance and suppress harmonic currents. In this paper, for the suppression of harmonic currents when the harmonic components of the grid voltage (third, fifth, and seventh harmonics) vary, an adaptive virtual harmonic resistance algorithm is proposed. It can adaptively adjust the size of the virtual harmonic resistance based on the changes in the harmonic content of the grid voltage. This adaptive approach improves the quality of the grid-connected current to meet the grid standards.

### 4.1. Adaptive Virtual Harmonic Resistor Implementation Method

In this paper, improving the quality of grid-connected currents and promoting sinusoidal waveform output when the grid voltage contains third, fifth, and seventh harmonic components are focused on by introducing a virtual impedance control strategy. Reference [18] discusses the selection of virtual impedance types and suggests that designing the virtual harmonic impedance as virtual harmonic resistance is more practical, since it provides damping effects on the harmonics. Virtual impedance can be categorized into three types: virtual resistance, virtual inductance, and virtual impedance R-L. To select the virtual impedance types, the virtual harmonic resistance and fundamental inductance need to be determined in this paper. The implementation method of adaptive virtual harmonic impedance consists of three main steps.

**Figure 5:**
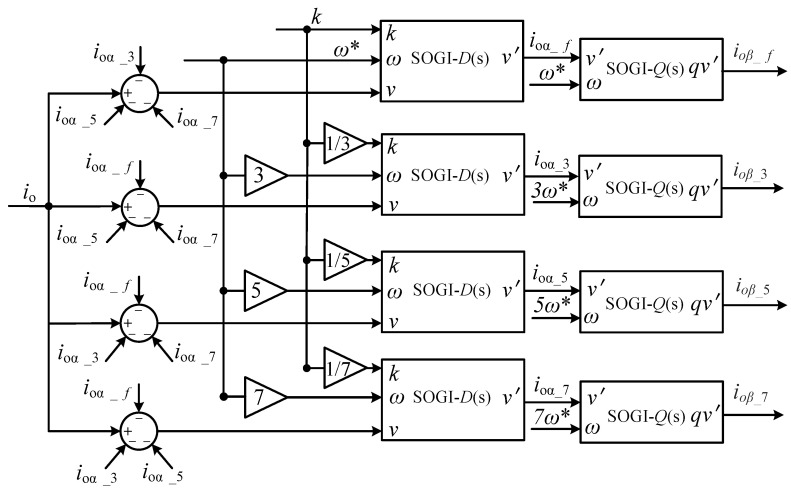
Block diagram of the signal separation structure.

**Figure 6:**
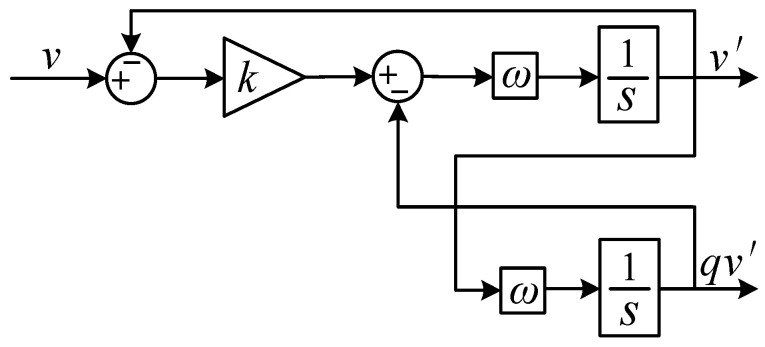
Second-order generalized integral structure.

Step (i): Separation of fundamental current and harmonic currents. As depicted in Figure 2, the implementation of adaptive virtual harmonic resistance requires separating the fundamental and harmonic currents from the grid-connected current. In this paper, a signal separation module based on multiple second-order generalized integrators (SOGIs) [26] is utilized to extract the fundamental and harmonic currents from the grid-connected current. The signal separation module decouples the harmonic signals using cross-feedback networks to isolate the interaction between different harmonics in the current signal. Then, using a second-order generalized integrator *D*(*s*), the fundamental and harmonic current components are separated on the α-axis at different frequencies; when *w* represents the fundamental and odd harmonic frequencies and kw is a constant value, it is possible to isolate the fundamental and harmonic currents. Finally, by utilizing a second-order generalized integrator *Q*(*s*), the fundamental and harmonic current components are obtained on the *β*-axis; when *w* represents the fundamental and odd harmonic frequencies, the *αβ*-axis components of the fundamental and harmonic currents can be constructed. The structure of the signal extraction is shown in Figure 5, and the second-order generalized integral structure is illustrated in Figure 6.

As shown in Figure 6, the transfer function of the SOGI can be obtained as follows:

(10) $$D(s)=\frac{v^{\prime }(s)}{v(s)}=\frac{kws}{s^{2}+kws+w^{2}}$$

(11) $$Q(s)=\frac{qv^{\prime }(s)}{v(s)}=\frac{kw^{2}}{s^{2}+kws+w^{2}}$$

where *k* is the damping adjustment coefficient, determining the bandwidth, and *w* is the resonance angular frequency, determining the center angular frequency. Equation (10) is primarily used for separating the fundamental and harmonic currents from the grid-connected current. In contrast to three-phase signals that can be transformed into *αβ*-axis components using the Clarke transformation, single-phase signals lack this capability. However, the SOGI shown in Equation (11) can obtain an input signal orthogonal to the input signal itself [27]. Therefore, it is possible to construct the *αβ*-axis components using the second-order generalized integral *Q*(*s*).

Step (ii): Extraction of fundamental and harmonic current magnitudes. To achieve the adaptive variation of virtual harmonic impedance, it is necessary to extract the amplitude of the fundamental and harmonic currents from the grid current, defining the output voltage as

(12) $$u_{c}=U_{m}\sin(wt)=U_{m}\sin\theta$$

The voltage signal is subjected to orthogonal transformation to obtain components on the *αβ*-axis.

(13) $$\left[\begin{array}{c} u_{c\alpha }\\ u_{c\beta } \end{array}\right]=\left[\begin{array}{c} U_{m}\sin wt\\ U_{m}\cos wt \end{array}\right]=\left[\begin{array}{c} U_{m}\sin\theta \\ U_{m}\cos\theta \end{array}\right]$$

**Figure 7:**
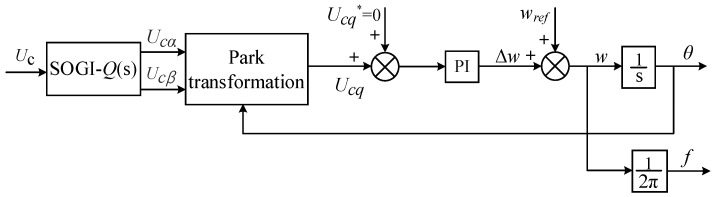
Block diagram of the SOGI-PLL structure.

The voltage signal is transformed using the Park transformation,

(14) $$\left[\begin{array}{c} u_{cd}\\ u_{cq} \end{array}\right]=\left[\begin{array}{cc} \sin\theta_{1} & \cos\theta_{1}\\ \cos\theta_{1} & -\sin\theta_{1} \end{array}\right]\left[\begin{array}{c} u_{c\alpha }\\ u_{c\beta } \end{array}\right]=\left[\begin{array}{c} \cos(\theta -\theta_{1})\\ sin(\theta -\theta_{1}) \end{array}\right]$$

where *θ*_1_ is the voltage measurement angle value obtained from the SOGI-PLL phase-locked loop output. When *U_cq_* = 0, it ensures that *θ* − *θ*_1_ equals zero, thereby guaranteeing that the measured angle value matches the actual value. Initially, the grid voltage is phase-locked using a second-order generalized integrator phase-locked loop (SOGI-PLL) [28], as illustrated in Figure 7, to obtain the fundamental angular frequency. Similarly, the angular frequencies of the third, fifth, and seventh harmonics can be obtained. The fundamental and harmonic currents on the *αβ* axis are then transformed into the *dq* axis through coordinate transformation. Based on the components on the *d* and *q* axes, the amplitudes of the fundamental and respective harmonic currents are determined.

Taking the extraction of the fundamental current amplitude as an example, the fundamental current extracted through the separation module can be denoted as *i_oα_f_* = *I_m_f_* cos(*wt* + *θ*). It can be inferred that the harmonic currents on the *β*-axis, obtained from the signal separation module, lag the fundamental current on the α-axis by 90 degrees, *i_oβ_f_* = *I_m_f_* sin(*wt* + *θ*). The transformation matrix *A* from the *αβ*-axis to the *dq*-axis is given by

(15) $$A=\left[\begin{array}{cc} \sin wt & -\cos wt\\ \cos wt & \sin wt \end{array}\right]$$

The transformation process of the fundamental current from the *αβ* axis to the *dq* axis coordinates is shown in Equation (16), as follows

(16) $$\begin{array}{ll} \left[\begin{array}{c} i_{od\_f}\\ i_{oq\_f} \end{array}\right] & =\left[\begin{array}{cc} \sin wt & -\cos wt\\ \cos wt & \sin wt \end{array}\right]\left[\begin{array}{c} i_{o\alpha \_f}\\ i_{o\beta \_f} \end{array}\right]\\ & =I_{m\_f}\left[\begin{array}{c} \cos(wt+\theta )\sin wt-\sin(wt+\theta )\cos wt\\ \cos(wt+\theta )\cos wt+\sin(wt+\theta )\sin wt \end{array}\right]\\ & =I_{m\_f}\left[\begin{array}{c} -\sin\theta \\ \cos\theta \end{array}\right] \end{array}$$

From Equation (16), we know that

(17) $$\sqrt{{\left(i_{od\_f}\right)}^{2}+{\left(i_{oq\_f}\right)}^{2}}=\sqrt{{\left(-I_{m\_f}\sin\theta \right)}^{2}+{\left(I_{m\_f}\cos\theta \right)}^{2}}=I_{m\_f}$$

Hence, by performing coordinate transformation, the amplitude of the fundamental current can be obtained. From Equation (17), it can be observed that the amplitude of the fundamental current is independent of the initial phase of the fundamental current. Similarly, the amplitudes of the third, fifth, and seventh harmonic currents can be obtained using the same approach.

**Figure 8:**
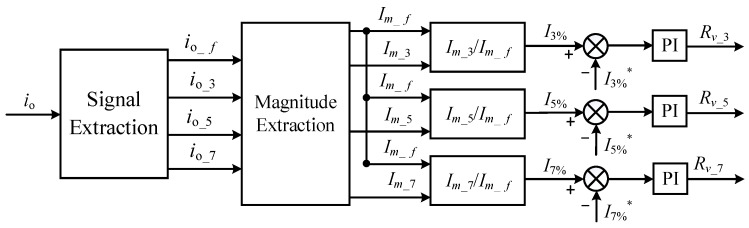
Block diagram for the construction of the adaptive virtual nth (*n* = 3,5,7) harmonic impedance.

Step (iii): Implementation of virtual harmonic resistors. To improve the quality of grid currents during variations in the harmonic content in grid voltages, it is necessary to achieve adaptive variations of the virtual harmonic impedance. This can be accomplished by introducing the harmonic content into the negative feedback control to achieve the desired objective. The block diagram of the adaptive virtual *n*th (*n* = 3,5,7) harmonic impedance construction is shown in Figure 8. First, the fundamental and harmonic currents are separated from the grid current. Then, the amplitude of the fundamental current and harmonic currents is extracted, and the *n*th (*n* = 3,5,7) harmonic content is obtained. Finally, the difference between the *n*th (*n* = 3,5,7) harmonic content and the reference value of the *n*th (*n* = 3,5,7) harmonic content is adjusted by a PI controller, and its output value represents the virtual *n*th (*n* = 3,5,7) harmonic impedance. The *n*th (*n* = 3,5,7) harmonic content is expressed by *I_n_*_%_, whose value is the ratio of the harmonic current amplitude to the fundamental current amplitude, and *I_n_*_%_* represents the reference value of the *n*th (*n* = 3,5,7) harmonic content. In Figure 6, *i_o_f_*, *i_o__*_3_, *i_o__*_5_ and *i_o__*_7_ represent the fundamental current, the third harmonic current, the fifth harmonic current, and the seventh harmonic current obtained through the signal separation module of the grid current io. *I_m_f_*, *I_m__*_3_, *I_m__*_5_ and *I_m__*_7_ denote the amplitudes of the fundamental current, the third harmonic current, the fifth harmonic current, and the seventh harmonic current obtained through the amplitude extraction module applied to the fundamental and harmonic currents.

The virtual nth (*n* = 3,5,7) harmonic impedance is obtained through PI regulation by taking the difference between the harmonic content *I_n_*_%_ and the reference value *I_n_*_%_*. The underlying principle is as follows: when the difference between the feedback *I_n_*_%_ and *I_n_*_%_* is greater than 0, the PI regulation increases the PI output. The PI output value represents the virtual nth harmonic impedance, and the voltage across the virtual nth harmonic impedance is introduced into the outer voltage loop through negative feedback. This is equivalent to serially connecting a virtual nth harmonic impedance in the harmonic equivalent circuit of the grid-connected system shown in Figure 4c. In the equivalent circuit of the grid-connected system, it acts as if the grid-side impedance is in series with the virtual nth harmonic impedance, increasing the magnitude of *Z_o_*(*s*) + *Z_g_*(*s*) and thus reducing the nth harmonic current while keeping the fundamental current unchanged. Consequently, the nth harmonic current decreases, leading to a reduction in the nth harmonic content. Through multiple feedback iterations, the difference between the feedback *I_n_*_%_ and *I_n_*_%_* approaches zero, ultimately achieving stability. As a result, the harmonic content approaches the reference value, thereby accomplishing the goal of adaptive improvement in the grid current quality.

### 4.2. Introduction of the Virtual Harmonic Impedance and Fundamental Reactance into the Equivalent Model

Based on the previous analysis, it is evident that the algorithm for adaptive virtual harmonic resistance is not applicable to virtual fundamental reactance. The design of the fundamental reactance parameters is primarily achieved through system stability analysis, as elaborated in Section 4.3. In this study, the algebraic virtual impedance method proposed in References [22,29] is employed to obtain the voltage across the virtual impedance. This voltage on the virtual impedance is then incorporated into the voltage–current dual-loop control to achieve regulation of the output current by the virtual impedance. The voltage on the fundamental and harmonic impedances on the *αβ* axis is expressed as follows:

(18) $$\left[\begin{array}{c} U_{\alpha \_f}\\ U_{\beta \_f} \end{array}\right]=\left[\begin{array}{cc} R_{v\_f} & -w^{*}L_{v\_f}\\ w^{*}L_{v\_f} & R_{v\_f} \end{array}\right]\left[\begin{array}{c} i_{o\alpha \_f}\\ i_{o\beta \_f} \end{array}\right]$$

(19) $$\left[\begin{array}{c} U_{\alpha \_3}\\ U_{\beta \_3} \end{array}\right]=\left[\begin{array}{cc} R_{v\_3} & -3w^{*}L_{v\_3}\\ 3w^{*}L_{v\_3} & R_{v\_3} \end{array}\right]\left[\begin{array}{c} i_{o\alpha \_3}\\ i_{o\beta \_3} \end{array}\right]$$

(20) $$\left[\begin{array}{c} U_{\alpha \_5}\\ U_{\beta \_5} \end{array}\right]=\left[\begin{array}{cc} R_{v\_5} & -5w^{*}L_{v\_5}\\ 5w^{*}L_{v\_5} & R_{v\_5} \end{array}\right]\left[\begin{array}{c} i_{o\alpha \_5}\\ i_{o\beta \_5} \end{array}\right]$$

(21) $$\left[\begin{array}{c} U_{\alpha \_7}\\ U_{\beta \_7} \end{array}\right]=\left[\begin{array}{cc} R_{v\_7} & -7w^{*}L_{v\_7}\\ 7w^{*}L_{v\_7} & R_{v\_7} \end{array}\right]\left[\begin{array}{c} i_{o\alpha \_7}\\ i_{o\beta \_7} \end{array}\right]$$

According to Section 4.1, the virtual fundamental inductance and virtual harmonic impedance are selected in this study. Therefore, in Equations (18)–(21), *R_v_f_*, *L_v_*__3_, *L_v_*__5_ and *L_v_*__7_ are all set to zero, while w* represents the fundamental angular frequency. By summing the components of each frequency on the *αβ* axis, the total voltage of the virtual impedance on the *αβ* axis can be obtained, as shown in Equation (22). The total voltage on the *αβ* axis is introduced into the voltage–current dual-loop control in the form of negative feedback. It is subtracted from the reference voltage of the power outer loop to simulate the influence of the actual impedance. The introduction of the voltage on the virtual impedance into the voltage–current dual-loop control does not involve differentiation operations, thereby avoiding the problem of amplifying high-frequency noise.

(22) $$\begin{array}{l} U_{z\alpha }=U_{\alpha \_f}+U_{\alpha \_3}+U_{\alpha \_5}+U_{\alpha \_7}\\ U_{z\beta }=U_{\beta \_f}+U_{\beta \_3}+U_{\beta \_5}+U_{\beta \_7} \end{array}$$

**Figure 9:**
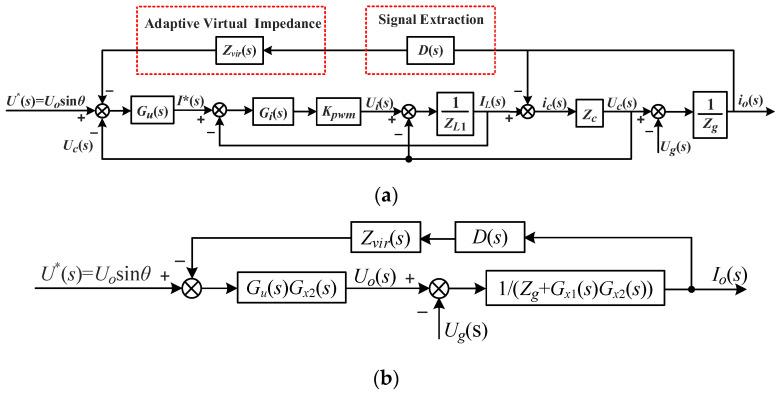
Voltage–current dual-loop equivalent model with the virtual impedance introduced: (**a**) original equivalent model and (**b**) simplified equivalent model.

The introduction of the voltage on the virtual impedance into the voltage–current dual-loop equivalent model is depicted in Figure 9a. The model can be simplified by moving the comparison and extraction points, as illustrated in Figure 9b, which shows a simplified equivalent model.

The open-loop transfer function of the system can be obtained from Figure 9 as

(23) $$G_{open}(s)=\frac{G_{u}(s)G_{x2}(s)D(s)Z_{vir}(s)}{Z_{g}+G_{x1}(s)G_{x2}(s)}$$

where G_*x*1_(*s*) and G*_x_*_2_(*s*) can be expressed by Equations (20) and (21)

(24) $$G_{x1}(s)=\frac{Z_{L1}+G_{i}(s)K_{pwm}}{G_{i}(s)K_{pwm}}$$

(25) $$G_{x2}(s)=\frac{G_{i}(s)K_{pwm}Z_{c}}{Z_{L1}+Z_{c}+G_{i}(s)K_{pwm}+G_{u}(s)G_{i}(s)K_{pwm}Z_{c}}$$

### 4.3. System Stability Analysis

The implementation of the adaptive virtual harmonic impedance and fundamental reactance requires first meeting the stability requirements of the system. The root locus plot can be generated based on the open-loop transfer function in Equation (23) to determine the parameter ranges for the virtual harmonic impedance and fundamental reactance variations. The stability of the system can be analyzed using the root locus plot.

**Figure 10:**
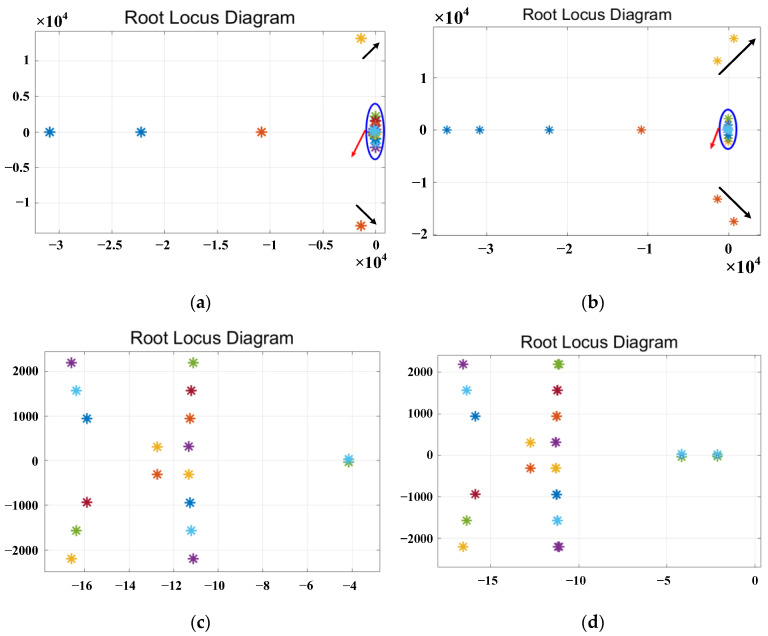
Root locus plot for varying the parameter of the virtual fundamental inductance. (**a**,**b**) The root locus plots near the stability boundary for *L_v_f_* in different parameter ranges. (**c**,**d**) The root locus plot magnified as indicated by the red arrow.

**Figure 11:**
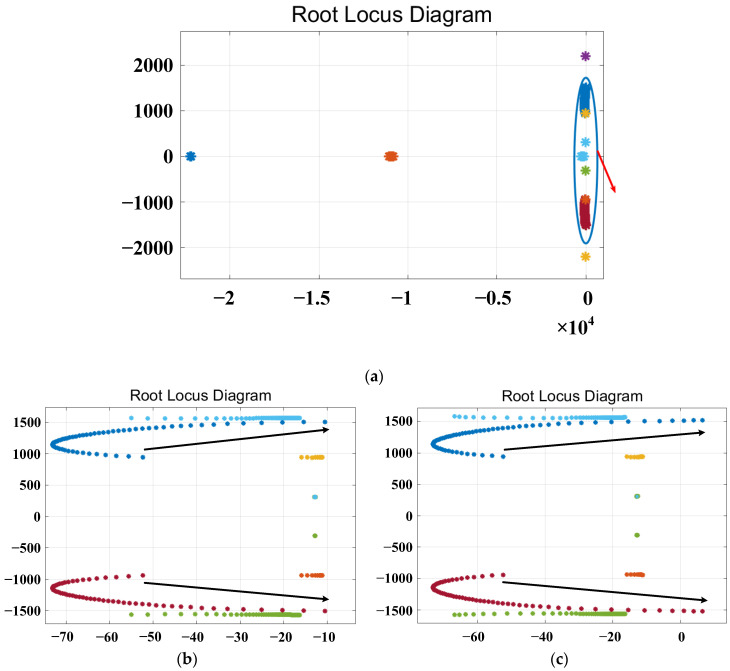
Root locus plot for varying the parameter of the virtual 3rd harmonic resistance. (**a**) The overall trend plot of the root locus. (**b**,**c**) The root locus curves magnified near the critical stability points for varying parameter ranges of *R_v_*__3_.

**Figure 12:**
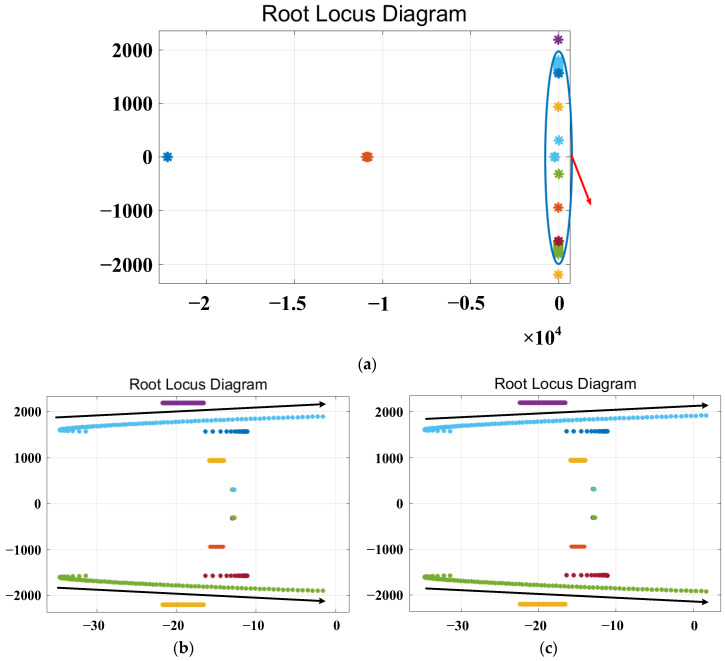
Root locus plot for varying the parameter of the virtual 5th harmonic resistance. (**a**) The overall trend plot of the root locus. (**b**,**c**) The root locus curves magnified near the critical stability points for varying parameter ranges of *R_v_*__5_.

**Figure 13:**
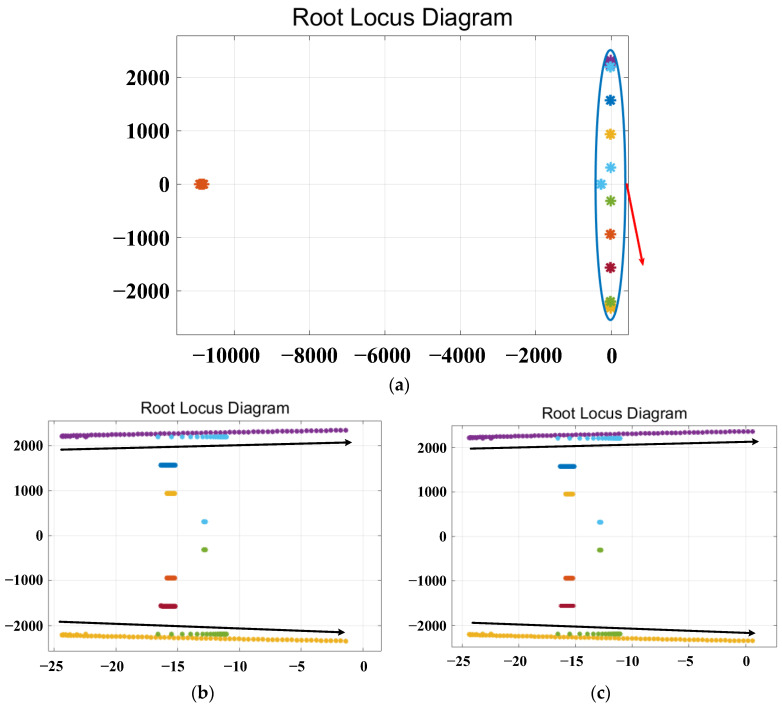
Root locus plot for varying the parameter of the virtual 5th harmonic resistance. (**a**) The overall trend plot of the root locus. (**b**,**c**) The root locus curves magnified near the critical stability points for varying parameter ranges of *R_v_*__7_.

The root locus plot with varying parameters *L_v_f_*, *R_v_*__3_, *R_v_*__5_ and *R_v_*__7_ is shown in Figure 10, Figure 11, Figure 12 and Figure 13, respectively.

The design of the fundamental impedance parameters should first satisfy the stability requirements of the system. Figure 10 shows the root locus plot with varying parameters of the virtual fundamental inductance *L_v_f_*, where the parameters are varied in integer form. Figure 10a,b represent the root locus plots with *L_v_f_* ranging from 0 to 1 mH and 0 to 2 mH, respectively. Figure 10c,d is an enlarged view of the critical region (indicated by the red arrow) near the stability boundary, different colored points represent different root locus plot. The root locus of the open-loop transfer function depicts the distribution of poles on the s-plane. When there are poles in the right half of the s-plane, the system is unstable. The black arrow indicates the direction in which the root locus changes. From Figure 10a, it can be observed that the poles changing in the direction of the black arrows do not cross the right-half plane. Similarly, Figure 10c indicates that there are no poles crossing the right-half plane near the critical point. The root locus of *L_v_f_* within the parameter range of 0–1 mH does not cross the right half-plane, indicating system stability. On the other hand, from Figure 10b, it can be observed that the poles changing in the direction of the black arrows cross the right-half plane. Similarly, Figure 10d indicates that there are no poles crossing the right-half plane near the critical point. The root locus for *L_v_f_* ranging from 0 to 2 mH partially crosses the right half-plane, indicating system instability. Based on the above analysis, it can be concluded that the system remains stable within the range of 0–1 mH for the virtual fundamental harmonic inductance *L_v_f_*.

Figure 11 illustrates the root locus plot with varying parameters of the virtual third harmonic resistor *R_v_*__3_, where the parameters are varied in integer form. Figure 11a shows the overall trend of the root locus curves, while Figure 11b,c display the root locus diagrams for *R_v_*__3_ within the ranges of 0–60 Ω and 0–65 Ω, respectively. The red arrow-indicated regions in Figure 11a are the magnified root locus curves. The black arrow indicates the direction in which the root locus changes. From Figure 11b, it can be observed that the poles changing in the direction of the black arrows do not cross the right-half plane. The root locus curves for *R_v_*__3_ within the parameter range of 0–60 Ω do not cross the right-half plane, indicating system stability. However, from Figure 11c, it can be observed that the poles changing in the direction of the black arrows cross the right-half plane. The root locus curves for *R_v_*__3_ within the range of 0–65 Ω cross the right-half plane, indicating system instability. Based on the above analysis, it can be concluded that the system remains stable within the range of 0–60 Ω for the virtual third harmonic resistor *R_v_*__3_.

Figure 12 shows the root locus plot with varying parameters of the virtual fifth harmonic resistor *R_v_*__5_, where the parameters are varied in integer form. Figure 12a shows the overall trend of the root locus, while Figure 12b,c present the root locus diagrams for *R_v_*__5_ within the ranges of 0–85 Ω and 0–90 Ω, respectively. The red arrow in Figure 12a indicates the magnified view of the root locus. The black arrow indicates the direction in which the root locus changes. From Figure 12b, it can be observed that the poles changing in the direction of the black arrows do not cross the right-half plane. The root locus for *R_v_*__5_ in the parameter range of 0–85 Ω does not cross the right-half plane, indicating system stability. However, from Figure 12c, it can be seen that the poles changing in the direction of the black arrows cross the right-half plane. The root locus for *R_v_*__5_ in the range of 0–90 Ω partially crosses the right-half plane, indicating system instability. Based on the above analysis, it can be concluded that the system remains stable within the range of 0–85 Ω for the virtual fifth harmonic resistor *R_v_*__5_.

Figure 13 illustrates the root locus plot with the virtual seventh harmonic resistor *R_v_*__7_ varying as parameters in integer form. Figure 13a represents the overall trend of the root locus, while Figure 13b,c display the root locus diagrams for *R_v_*__7_ within the ranges of 0–70 Ω and 0–75 Ω, respectively. The red arrow in Figure 13a indicates the magnified view of the root locus. The black arrow indicates the direction in which the root locus changes. From Figure 12b, it can be observed that the poles changing in the direction of the black arrows do not cross the right-half plane. The root locus for *R_v_*__7_ in the parameter range of 0–70 Ω does not cross the right-half plane, indicating system stability. However, from Figure 12c, it can be seen that the poles changing in the direction of the black arrows cross the right-half plane. The root locus for *R_v_*__7_ in the parameter range of 0–75 Ω partially crosses the right-half plane, indicating system instability. Based on the above analysis, it can be concluded that the system remains stable within the range of 0–70 Ω for the virtual seventh harmonic resistor *R_v_*__7_.

From the above analysis, we see that it is necessary to limit the virtual nth (*n* = 3,5,7) harmonic impedance to ensure system stability. The virtual fundamental virtual inductance is restricted to the range of 0–1 mH, and the virtual third, fifth, and seventh harmonic impedances are limited to 0–60 Ω, 0–85 Ω, and 0–70 Ω, respectively. 

**Figure 14:**
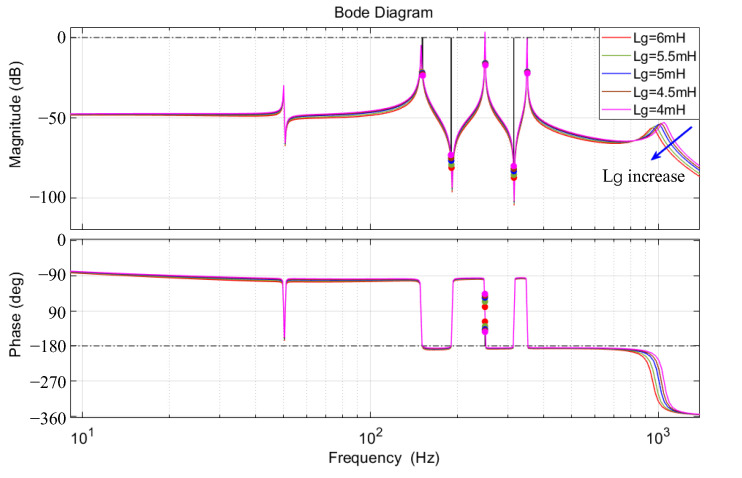
Bode plot of the open-loop transfer function when the line inductance increases.

The open-loop transfer function expression shown in Equation (23) does not include the grid voltage; hence, the variation in grid harmonic content is irrelevant to system stability. This paper considers the stability analysis when there are slight changes in the line impedance. The virtual fundamental virtual inductance is set to 1 mH, and the virtual third, fifth, and seventh harmonic impedances are set to 60 Ω, 85 Ω, and 70 Ω, respectively. In the simulation and experimental parameters, the line inductance is 5 mH. Based on Equation (23), the Bode plot of the open-loop transfer function when the line inductance increases is shown in Figure 14.

As can be seen from Figure 14, when *L*_g_ = 5 mH, the amplitude margin and phase angle margin are both positive, indicating that the system is stable at this time. When *L*_g_ is slightly reduced, the amplitude margin and phase angle margin are greater than 0, and the system remains stable. When *L*_g_ is slightly increased, the amplitude margin and phase angle margin are greater than 0, and the system remains stable. It can be seen that the system can maintain stability when the line impedance is slightly disturbed.

The combination of an LC filter with the line impedance can form an LCL-type filter, which may introduce resonance peaks and potentially lead to system instability. This issue can be addressed by employing the active damping method through capacitor current feedback [30].

## 5. Simulation and Experimental Results

In this section, the effectiveness of the harmonic current suppression strategy based on the adaptive harmonic virtual resistance was validated through simulations and experimental results. The detailed system parameters are listed in Table 2.

### 5.1. Simulation Results

To simulate the impact of the harmonic voltages on the grid current quality, third, fifth, and seventh harmonics were injected into the grid voltage using MATLAB/Simulink. Two operating conditions were designed. Condition 1 included a background harmonic with 8% of the third harmonic, 7% of the fifth harmonic, and 5% of the seventh harmonic. Condition 2 involved a change in the harmonic content of the grid voltage after 2 s, with 10% of the third harmonic, 8% of the fifth harmonic, and 7% of the seventh harmonic. The reference value of the third harmonic content is set to *I*_3%_* = 2.0%, the reference value of the fifth harmonic content is set to *I*_5%_* = 1.7%, and the reference value of the seventh harmonic content is set to *I*_7%_* = 1.5%.

**Figure 15:**
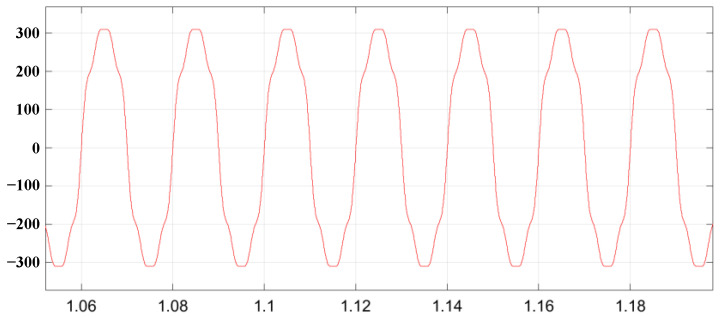
Voltage waveform under the grid voltage distortion.

**Figure 16:**
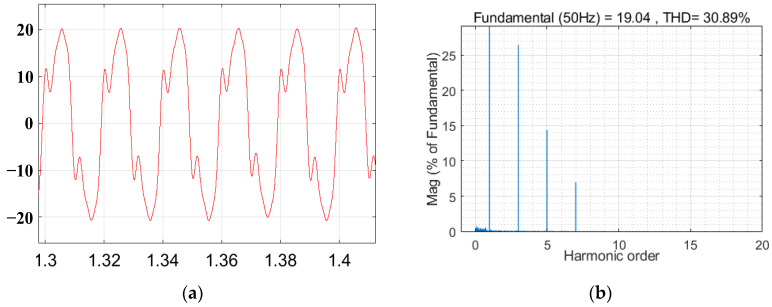
Grid current waveform and THD without the inclusion of the virtual harmonic impedance under distorted grid voltages. (**a**) The grid current waveform. (**b**) THD of the grid currents.

**Figure 17:**
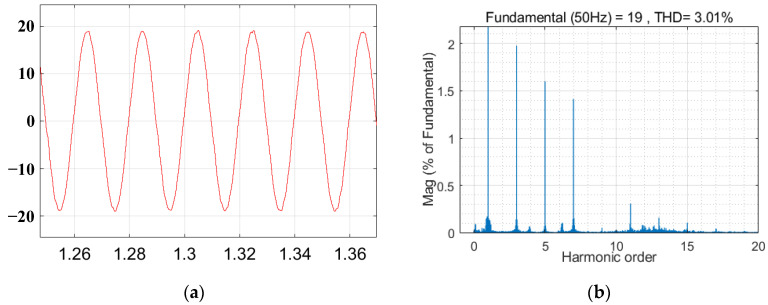
Waveform of the grid current and the THD of the grid current when adaptive virtual harmonic impedance is introduced under Condition 1. (**a**) The grid current waveform. (**b**) THD of the grid currents.

**Figure 18:**
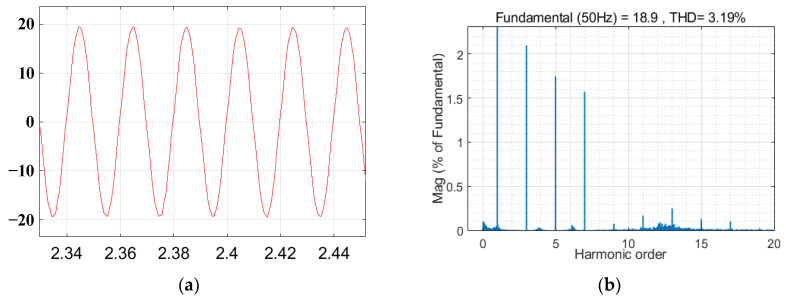
Waveform of the grid current and the THD of the grid current when the adaptive virtual harmonic impedance is introduced under Condition 2. (**a**) The grid current waveform. (**b**) THD of the grid currents.

The voltage waveform under grid voltage distortion is shown in Figure 15. The grid current waveform and total harmonic distortion (THD) without the inclusion of the virtual harmonic impedance under distorted grid voltages are illustrated in Figure 16. The grid current waveform and total harmonic distortion with the inclusion of virtual harmonic impedance under Condition 1 and Condition 2 are shown in Figure 17 and Figure 18, respectively. Based on the simulation results from Figure 16, Figure 17 and Figure 18, the harmonic content and THD of the grid current under different scenarios are presented in Table 3.

Furthermore, Figure 15 shows that the presence of harmonics in the grid voltage leads to distortions in the grid current, significantly impacting the quality of the grid current and preventing it from meeting the grid standards. The comparison between Figure 16, Figure 17 and Figure 18 reveals that the incorporation of an adaptive virtual harmonic resistor effectively reduces the THD of the grid-connected current and enhances the waveform quality. In Condition 1, as observed from the comparison of Figure 16 and Figure 17, the introduction of an adaptive virtual resistor results in a significant reduction in the content of the third harmonic from 26.3% to 1.97%, the fifth harmonic from 14.2% to 1.61%, and the seventh harmonic from 6.8% to 1.43%. The levels of the third, fifth, and seventh harmonics are closely aligned with the set reference values of *I*_3%_* = 2.0%, *I*_5%_* = 1.7% and *I*_7%_* = 1.5%, indicating a small margin of error. In the case of Condition 2, the third, fifth, and seventh harmonic contents are *I*_3%_ = 2.08%, *I*_5%_ = 1.74% and *I*_7%_ = 1.54%, respectively. These values exhibit minimal deviation from the set harmonic content reference values. The detailed comparison of grid current harmonic content and total harmonic distortion (THD) under different operating conditions is presented in Table 3. By comparing Figure 17 and Figure 18, it can be observed that the adaptive harmonic virtual resistor can effectively improve the quality of the grid-connected current in the presence of increased background harmonics in the grid voltage.

### 5.2. Experimental Results

**Figure 19:**
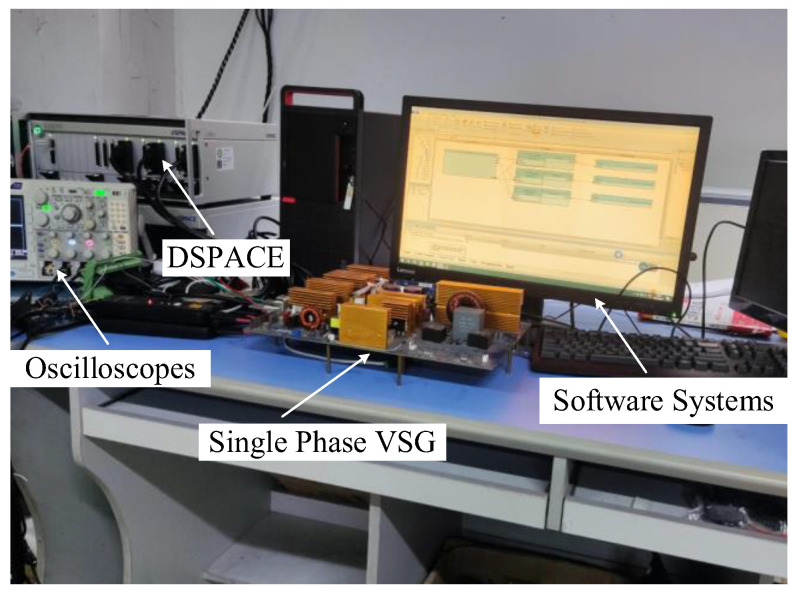
Experimental platform.

In this section, the effectiveness of the harmonic current suppression strategy based on the adaptive harmonic virtual resistors was further validated using the DSPACE SCALEXIO experimental platform (DSPACE, Paderborn, Germany). The parameters of each component in the system and the main parameters are consistent, as shown in Table 2. The experimental platform of the 1.5 kW single-phase voltage-source inverter is set up in this paper, as shown in Figure 19. A 14-bit analog-to-digital converter (ADC DS6221) (DSPACE) was used to sample the output voltage, inductance current and grid-connected current signals at a sampling frequency of 20 kHz. The I/O board (DS6202) (DSPACE) is configured as a PWM module with a switching frequency of 20 kHz. The voltage and current signals that need to be used in the control structure in the main circuit topology are collected by the ADC sampling module, and the sampled signals are sent to the DSPACE controller. PWM signal generated by DSPACE controller is used to control the single-phase voltage source grid-connected inverter. The output digital current and voltage signals are converted into analog signals by the DAC conversion module, and the output grid-connected current and current signal waveform are observed by oscilloscope (YOKOGAWA-DLM2024) (YOKOGAWA, Tokyo, Japen). The THD of the grid-connected current under different conditions is presented in Table 4. To simulate the impact of the background harmonics on the quality of the grid-connected current, 8% of the third harmonic, 7% of the fifth harmonic, and 5% of the seventh harmonic were injected into the grid voltage.

**Figure 20:**
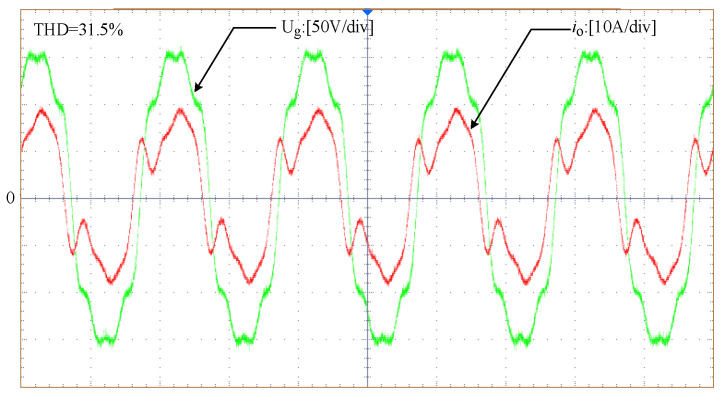
Grid voltage and grid current waveforms without an adaptive virtual harmonic resistor under full-load steady-state conditions.

**Figure 21:**
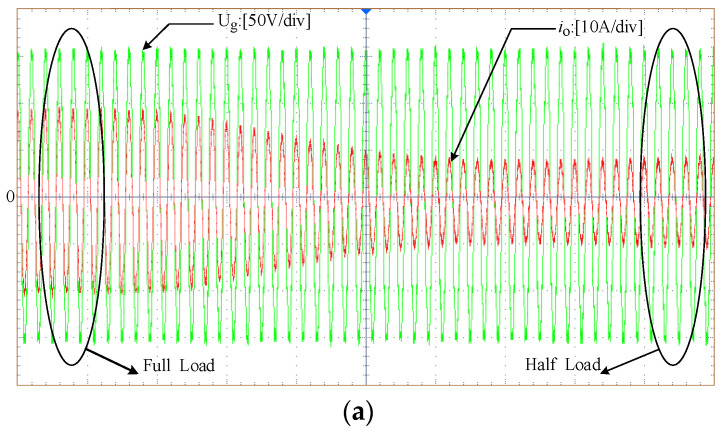

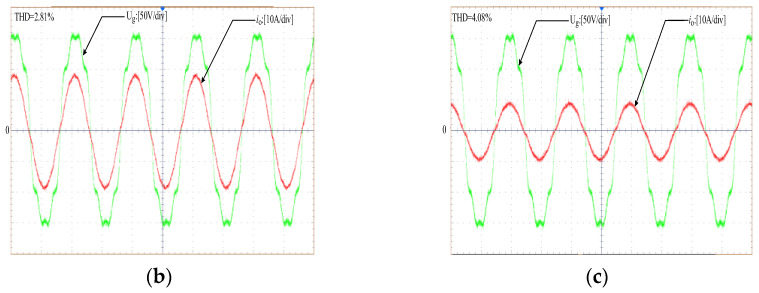
Grid voltage and grid current waveforms when transitioning from full-load steady-state to half-load steady-state with the introduction of an adaptive virtual harmonic resistor. (**a**) The overall trend when transitioning from full-load steady-state to half-load steady-state. (**b**) Full-load steady state. (**c**) Half-load steady state.

Figure 20 illustrates the grid voltage and grid current waveforms without an adaptive virtual harmonic resistor in a full-load steady state. The grid current waveform exhibits significant distortion, and it does not comply with the grid requirements. In contrast, Figure 21 shows the grid voltage and grid current waveforms with the introduction of an adaptive virtual harmonic resistor during the transition from the full-load steady state to half-load steady state. The grid current waveform exhibits a higher sinusoidal content and better waveform quality. It can be concluded that the addition of an adaptive virtual harmonic resistor can suppress harmonic currents and improve the quality of the grid-connected current.

This experiment only separated the fundamental, third, fifth, and seventh harmonic currents. Due to the lower content of higher-order harmonics and the increased computational effort required for their separation, harmonics above the seventh order were not separated. To further reduce harmonic content, it is necessary to isolate higher-order harmonics, which also represents a limitation of this method. The conclusion section has been added.

## 6. Conclusions

In this paper, control strategies are investigated to improve the grid-connected currents in single-phase grid-tied inverters operating in weak power grids. First, the mechanism of harmonic current generation is explained. Then, an inhibitory strategy using adaptive virtual harmonic resistors is proposed, highlighting the implementation method of adaptive virtual harmonic resistors. Stability analysis of the system is performed by examining the root locus. Finally, the effectiveness of the proposed adaptive virtual harmonic resistor strategy is validated through simulation and experimental results, demonstrating an improvement in the grid-connected current quality.

However, when the proposed adaptive virtual impedance method is implemented for high-order harmonic current compensation, the strategy needs to separate each harmonic current and construct the adaptive virtual harmonic impedance, which increases the complexity of the system and the calculation burden of the control system. Therefore, on the premise of meeting the quality of grid-connected current, this paper only carries out the impedance reshaping method on the low harmonic current to reduce the low harmonic current content. How to further reduce the high harmonic current without significantly increasing the calculation amount will be a future research direction.

## Figures and Tables

**Table 1:** Comparison of various control techniques.

Various Control Methods	Adaptive Harmonic Current Suppression	Computational Algorithm Burden	PCC Voltage	Limitation
Reference [11]	No	Medium	Do not need	May cause instability of the system
References [12,13]	No	High	Do not need	Poor dynamic performance
Reference [14]	No	High	Do not need	High complexity of system
Reference [15]	No	Media	need	Poor suppression of higher-order harmonics
References [16,17]	No	High	need	Amplification of high-frequency signal interference
Reference [18]	No	Medium	Do not need	Complexity of high-order harmonic suppression algorithms
Reference [19]	No	Media	Do not need	Complex algorithm
Reference [20]	No	Medium	need	May cause instability of the system
Reference [21]	No	High	Do not need	Complex algorithm
Proposed in this paper	Yes	Medium	Do not need	Complexity of high-order harmonic suppression algorithms

**Table 2:** System parameters.

Parameters	Value	
	Simulation	Experiment
DC voltage *U_dc_*	400 V	400 V
Fundamental voltage amplitude *U_g_*	314 V	155.5 V
Inverter-side filter inductor *L*_1_	4 mH	4 mH
Switching frequency *f_sw_*	20 kHz	20 kHz
Grid-side filter inductor and parasitic resistance *L*_g_ + *R*_g_	0.4 Ω + 5 mH	0.4 Ω + 5 mH
Filter capacitor *C*	15 uF	15 uF
Virtual fundamental inductor *L_v_f_*	0.5 mH	0.5 mH
Output power *P*	-	1.5 kW
Active power reference *P_ref_*	3 kW	-
Reactive power reference *Q_ref_*	0 var	-

**Table 3:** The harmonic content and THD of the grid current under different scenarios.

	3rd Harmonic Content	5th Harmonic Content	7th Harmonic Content	THD of the Grid Current
Without the addition of virtual harmonic impedance	26.3%	14.2%	6.8%	30.89%
Condition 1	1.97%	1.61%	1.43%	3.01%
Condition 2	2.08%	1.74%	1.54%	3.19%

**Table 4:** The THD of the grid-connected current under different situations.

Different Conditions	THD of the Grid Current
Under full-load conditions without the inclusion of the adaptive virtual impedance	31.5%
Under full-load conditions with the inclusion of the adaptive virtual impedance	2.81%
Under half-load conditions with the inclusion of the adaptive virtual impedance	4.08%

## Data Availability

The data that support the findings of this study are available from the corresponding author upon reasonable request.
